# Atrial Natriuretic Peptide Stimulates Dopamine Tubular Transport by Organic Cation Transporters: A Novel Mechanism to Enhance Renal Sodium Excretion

**DOI:** 10.1371/journal.pone.0157487

**Published:** 2016-07-08

**Authors:** Nicolás M. Kouyoumdzian, Natalia L. Rukavina Mikusic, María C. Kravetz, Brenda M. Lee, Andrea Carranza, Julieta S. Del Mauro, Marcela Pandolfo, Mariela M. Gironacci, Susana Gorzalczany, Jorge E. Toblli, Belisario E. Fernández, Marcelo R. Choi

**Affiliations:** 1 Cardiological Research Institute, National Scientific and Technical Research Council, Buenos Aires, Argentina; 2 Pathophysiology and Clinical Biochemistry Institute, Buenos Aires, Argentina; 3 Department of Pharmacology, School of Pharmacy and Biochemistry, University of Buenos Aires, Buenos Aires, Argentina; 4 Department of General Surgery, Johns Hopkins Hospital, Baltimore, Maryland, United States of America; 5 Department of Biological Chemistry, School of Pharmacy and Biochemistry, University of Buenos Aires, Buenos Aires, Argentina; 6 Deutsch Hospital, Buenos Aires, Argentina; 7 Department of Anatomy and Histology, School of Pharmacy and Biochemistry, University of Buenos Aires, Buenos Aires, Argentina; 8 Department of Pathophysiology, School of Pharmacy and Biochemistry, University of Buenos Aires, Buenos Aires, Argentina; University of Geneva, SWITZERLAND

## Abstract

The aim of this study was to demonstrate the effects of atrial natriuretic peptide (ANP) on organic cation transporters (OCTs) expression and activity, and its consequences on dopamine urinary levels, Na^+^, K^+^-ATPase activity and renal function. Male Sprague Dawley rats were infused with isotonic saline solution during 120 minutes and randomized in nine different groups: control, pargyline plus tolcapone (P+T), ANP, dopamine (DA), D-22, DA+D-22, ANP+D-22, ANP+DA and ANP+DA+D-22. Renal functional parameters were determined and urinary dopamine concentration was quantified by HPLC. Expression of OCTs and D1-receptor in membrane preparations from renal cortex tissues were determined by western blot and Na^+^, K^+^-ATPase activity was determined using in vitro enzyme assay. ^3^H-DA renal uptake was determined in vitro. Compared to P+T group, ANP and dopamine infusion increased diuresis, urinary sodium and dopamine excretion significantly. These effects were more pronounced in ANP+DA group and reversed by OCTs blockade by D-22, demonstrating that OCTs are implied in ANP stimulated-DA uptake and transport in renal tissues. The activity of Na^+^, K^+^-ATPase exhibited a similar fashion when it was measured in the same experimental groups. Although OCTs and D1-receptor protein expression were not modified by ANP, OCTs-dependent-dopamine tubular uptake was increased by ANP through activation of NPR-A receptor and protein kinase G as signaling pathway. This effect was reflected by an increase in urinary dopamine excretion, natriuresis, diuresis and decreased Na^+^, K^+^-ATPase activity. OCTs represent a novel target that links the activity of ANP and dopamine together in a common mechanism to enhance their natriuretic and diuretic effects.

## Introduction

The renal dopaminergic system is a local independent natriuretic system that contributes to preserving the normal balance of sodium and water, blood pressure levels and renal redox steady state [1]. Renal dopamine production results from decarboxylation of its precursor L-dopa, an enzymatic step which depends on L-dopa decarboxylase activity (also called aromatic acid decarboxylase: AADC) [2]. It has been demonstrated that proximal tubules represent the main source of renal dopamine, since this site exhibits a high concentration of AADC [3]. Several studies have proposed two L-aminoacid transporters namely type 1 and 2 (LAT-1 and LAT-2) as the transporters implicated in the uptake of the precursor L-dopa by the proximal tubular cells [4]. In addition, other non-neuronal transporters have been postulated to be the primary means of transport dopamine at the same location. In this sense, renal organic transporters are members of the group SLC22A (solute carrier superfamily), which includes the polyspecific organic cation transporters: OCT-1, OCT-2 and OCT-3 located mainly at the basolateral membrane of proximal tubules cells, and OCTN-1, OCTN-2 and OCTN-3 located mainly at the apical side of the proximal tubules cells [5–7].

As a major regulator of proximal tubule salt and water reabsorption, renal dopamine exerts its physiological actions through two families of receptors located at the tubular cell surface: D1-like receptors (D1R and D5R) and D2-like receptors (D2R, D3R and D4R) [8]. The importance of dopamine as a natriuretic hormone is reflected by its capacity to inhibit sodium transporters, specially the activity of Na^+^, K^+^-ATPase, in almost the entire nephron [9].

Atrial natriuretic peptide (ANP), discovered by de Bold, is a 28-amino-acid peptide synthesized and stored in the atrial myocytes and released in response to cardiac wall stretching or after endothelin and α-adrenergic stimulation [10]. Natriuretic effects of ANP are exerted through enhanced glomerular filtration rate and inhibition of sodium tubular reabsorption via direct and indirect mechanisms [11,12]. Based on the observation that dopamine and ANP share similar physiological effects, it has been proposed the existence of a possible interaction between natriuretic peptide hormones and the renal dopaminergic system. It has been reported that part of the inhibitory effects of ANP on sodium and water reabsorption are dependent on dopaminergic mechanisms, particularly those involving dopamine receptors and activity of Na^+^, K^+^-ATPase [11,13,14]. These findings allow us to hypothesize that ANP might modulate renal dopamine transport by the OCTs, therefore affecting its availability to interact with dopaminergic receptors. Through this mechanism both systems could interact synergistically, enhancing their natriuretic and diuretic effects. Therefore, the aim of this study is to demonstrate *in vivo* and *in vitro* the effects of ANP infusion on OCTs expression and activity, respectively, and its consequence on dopamine urinary levels, Na^+^, K^+^-ATPase activity and renal function.

## Materials and Methods

### Animal Protocol

Male Sprague Dawley rats weighing 300–350 g (from the Pathophysiology Department, School of Pharmacy and Biochemistry of University of Buenos Aires) were used following international guiding principles and local regulations regarding the care and use of laboratory animals for biomedical research as well as the “International Ethical Guiding Principles for Biomedical Research on Animals” established by the CIOMS (Council for International Organizations of Medical Sciences). The protocol was approved by the Institutional Committee for Care and Use of Laboratory Animals of the School of Pharmacy and Biochemistry of University of Buenos Aires (Permit Number: 2100–15; 0035638/15). Animals were housed in cages with a 12-hour light/dark cycle under conditions of controlled temperature and humidity. All animals had free access to water and standard chow (Commercial Rodents Purina Chow; Cooperation SRL, Argentina) until the day of the experiment. All surgery was performed under ethyl urethane anesthesia and all efforts were made to minimize suffering.

### Drugs

The following drugs were used in the experiments: rat atrial natriuretic peptide 1–28 (ANP) (American peptides Inc., CA, USA); ^3^H-dopamine, 28.0 Ci/mmol specific activity (New England Nuclear, Boston, Mass., USA); nomifensine; decynium 22 (D-22); benserazide; pargyline; tolcapone; dopamine; imidazol; ATP (adenosine 5′ triphosphate); bovine seroalbumin fraction V of Cohn; ouabain (Sigma-Aldrich Inc., St. Louis, Mo., USA); Folin Ciocalteus phenol reagent (Merck Co., USA) and EcoLite, a liquid scintillation (ICN Pharmaceutical Inc., Irvine, CA, USA). Standard Krebs bicarbonate (SKB) solution of the following composition (mM) was employed as incubation medium: 118 NaCl; 4.7 KCl; 1.2 MgSO_4_.7H_2_O; 1.0 NaH_2_PO_4_; 2.4 CaCl_2_; 0.004 EDTA; 11.1 glucose; 0.11 ascorbic acid and 26.0 NaHCO_3_.

### In vivo protocol

In order to minimize the confounding effects of endogenous dopamine, benserazide (an inhibitor of dopamine synthesis) was administrated to rats 24 and 2 hours before treatment (200 μg/kg, i.p.). Rats were anesthetized with 10% w/v ethyl urethane (1.2 g/kg, i.p.) and placed on a thermostatically controlled heating pad to keep body temperature at 37°C. A tracheotomy was performed and a PE-90 tube (3 cm long) was inserted into the trachea to maintain an open airway. The left femoral vein was cannulated with a Silastic cannula (0.12 mm i.d.) for continuous infusion. The right carotid artery was catheterized with a T4 tube for blood sampling and arterial pressure monitoring, using a Statham GOULD P23ID transducer coupled to a Grass Polygraph 79D. The bladder was cannulated with a PE-75 cannula for urine collection. During a stabilization period of 60 minutes, the animals were infused with 0.15 M NaCl isotonic saline solution (ISS) at a rate of 3.0 mL/h (Syringe Infusion Pump, SageTM, Orion) to reach a steady state in diuresis and allow urine collection (baseline period). After this period, the animals were infused at the same rate with ISS plus different drugs (which are stated below) for 2 hours (experimental period). To determine renal functional parameters, urine and blood samples were collected every 30 and 60 minutes, respectively. Mean arterial pressure (MAP) was recorded every 30 minutes (Table 1).

**Table 1:** In vivo experimental protocol.

Experimental group	30-minute time periods							
	Stabilizationperiod (0–30 min)	Stabilization period (30–60 min)	E1(basal)	E2(basal)	E3(0–30 min)	E4(30–60 min)	E5(60–90 min)	E6(90–120 min)
**Control**	Vehicle	Vehicle	Vehicle	Vehicle	Vehicle	Vehicle	Vehicle	Vehicle
**P+T**	Vehicle	Pargyline and Tolcapone + Vehicle	Vehicle	Vehicle	Vehicle	Vehicle	Vehicle	Vehicle
**ANP**	Vehicle	Pargyline and Tolcapone + Vehicle	Vehicle	Vehicle	ANP	ANP	ANP	ANP
**DA**	Vehicle	Pargyline and Tolcapone + Vehicle	Vehicle	Vehicle	Dopamine	Dopamine	Dopamine	Dopamine
**ANP plus DA**	Vehicle	Pargyline and Tolcapone + Vehicle	Vehicle	Vehicle	ANP plus Dopamine	ANP plus Dopamine	ANP plus Dopamine	ANP plus Dopamine
**D-22**	Vehicle	Pargyline and Tolcapone + Vehicle	D-22	D-22	D-22	D-22	D-22	D-22
**ANP plus D-22**	Vehicle	Pargyline and Tolcapone + Vehicle	D-22	D-22	ANP plus D-22	ANP plus D-22	ANP plus D-22	ANP plus D-22
**DA plus D-22**	Vehicle	Pargyline and Tolcapone + Vehicle	D-22	D-22	Dopamine plus D-22	Dopamine plus D-22	Dopamine plus D-22	Dopamine plus D-22
**ANP plus DA plus D-22**	Vehicle	Pargyline and Tolcapone + Vehicle	D-22	D-22	ANP, Dopamine plus D-22	ANP, Dopamine plus D-22	ANP, Dopamine plus D-22	ANP, Dopamine plus D-22

Vehicle: Isotonic Saline Solution; Pump rate: 3,0 ml/h

Experimental periods (E1, E2, E3, E4, E5 and E6): urine collection

E2, E4, E6: blood pressure measurement and blood collection

Drugs doses: Pargyline (20 mg/kg, i.v.); Tolcapone (300 μg/kg, i.v.); D-22 (10 μg/kg/h); ANP (10 μg/kg, i.v. bolus plus 20 μg/kg/h); Dopamine (100 μg/kg/h).

The animals were randomized into the following experimental groups:

1. Control group (C) (n = 8): infused with ISS for 120 minutes (experimental period).
  With the exception of the control group, all groups received an i.v. bolus of pargyline (20 mg/kg) and tolcapone (300 μg/kg) dissolved in 0.1 ml of ISS, inhibitors of the dopamine catabolic enzymes monoaminoxidase (MAO) and cathecol-O-methyl-transferase (COMT) respectively, 30 minutes before the beginning of the experimental period in order to prevent the catabolism of the exogenous dopamine infused.
2. Pargyline + tolcapone (P+T) (n = 6): animals were infused with ISS for 120 minutes.
3. ANP (n = 7): animals received ANP (5 μg/kg, dissolved in 0.1 ml of ISS) in i.v. bolus at the beginning of the experimental period. Thereafter, rats were infused with ISS plus ANP (10 μg/kg/h) for 120 minutes.
4. Dopamine (n = 7): animals were infused with ISS plus dopamine (100 μg/kg/h) for 120 minutes.
5. D-22 (n = 6): D-22, a specific inhibitor of OCTs, was infused one hour before the experimental period at 10 μg/kg/h. Thereafter, animals were infused with ISS plus D-22 (10 μg/kg/h) for 120 minutes.
6. Dopamine + D-22 (n = 6): D-22 was infused one hour before the experimental period at 10 μg/kg/h. Thereafter, animals were infused with ISS plus dopamine (100 μg/kg/h) and D-22 (10 μg/kg/h) for 120 minutes.
7. ANP + D-22 (n = 6): D-22 was infused one hour before the experimental period at 10 μg/kg/h. An i.v. bolus of ANP (5 μg/kg) at the beginning of the experimental period was followed by ISS infusion plus ANP (10 μg/kg/h) and D-22 (10 μg/kg/h) for 120 minutes.
8. ANP + Dopamine (n = 6): animals received ANP (5 μg/kg) in i.v. bolus at the beginning of the experimental period, followed by ISS infusion plus ANP (10 μg/kg/h) and dopamine (100 μg/kg/h) for 120 minutes.
9. ANP + Dopamine + D-22 (n = 6): D-22 was infused one hour before the experimental period at 10 μg/kg/h. An i.v. bolus of ANP (5 μg/kg) at the beginning of the experimental period was followed by ISS infusion plus ANP (10 μg/kg/h), dopamine (100 μg/kg/h) and D-22 (10 μg/kg/h) for 120 minutes.

### Urine and blood measurements

To evaluate renal functionality, the following parameters were determined: urine and plasma sodium and creatinine (UNa, PLNa, UCr and PLCr, respectively), glomerular filtration rate (GFR) estimated by creatinine clearance (CrCl), fractional and urinary sodium excretion (FENa and UNa.UV, respectively) and urine flow rate (UV). UNa, PLNa, UCr and PLCr were measured by standard methods using an autoanalyzer. FENa, UNa.UV and CrCl were calculated according to standard formula. UV is expressed as μL/min, UNa and PLNa as mEq/L, FENa as the percentage (%) of filtered sodium, UNa.UV as μEq/min and CrCl as mL/min. To determine urinary dopamine concentration by HPLC, a fraction of urine was collected at the end of the experimental period into polyethylene tubes containing 100 μl of 6 N HCl.

### Western blot analysis for OCTs and D1 Receptor

Western blot analysis was performed to evaluate ANP effects on total amount of OCTs and D1 receptor, by using membrane preparations. Right kidney from each animal was removed and renal cortex was immediately dissected and separated under refrigeration. In order to isolate cellular membranes, additional sets of tissue samples were homogenized on ice with a Tissue Tearor (Biospec Products Inc), 1:9 weight/volume, in a buffer mixture: 50 mmol/L Tris and 300 mmol/L sucrose, pH 7.40, and centrifuged at 4000 rpm and 4°C during 15 minutes. Supernatants were centrifuged at 17000 rpm and 4°C during 45 minutes. Pellets were suspended in buffer containing 50 mmol/L Tris, 0.1 mmol/L EDTA, 0.1 mmol/L EGTA, 1% Triton-X-100, 1 mmol/L PMSF, 1 μmol/L pepstatin, and 2 μmol/L leupeptin, 1x protease inhibitor cocktail (Roche Diagnostics), and afterwards, samples were centrifuged at 12000 rpm during 20 minutes. Protein concentration in the Triton-soluble supernatant was determined by Lowry technique [15]. Samples of membrane preparation, containing similar amounts of protein (100 μg protein/lane), were separated by electrophoresis in 10% SDS-polyacrylamide gels (Bio-Rad Labs Inc) and then transferred to a PVDF membrane (Bio-Rad Labs Inc) and incubated with goat polyclonal anti-OCT-1 (Santa Cruz Biotechnology, Inc. 1:200 dilution), goat polyclonal anti-OCT-2 (Santa Cruz Biotechnology, Inc. 1:400 dilution), goat polyclonal anti-OCT-3 (Santa Cruz Biotechnology, Inc. 1:200 dilution) or goat polyclonal anti-D1R (Santa Cruz Biotechnology, Inc. 1:400 dilution). A secondary immunoreaction was performed with a biotinilated donkey anti-goat IgG (Santa Cruz Biotechnology, Inc. 1:1500 dilution), followed by a third step using streptavidin conjugated with horseradish peroxidase (GE Healthcare Life Sciences; dilution of 1:2000). The samples were revealed by chemiluminescence using ECL reagent (Amersham Pharmacia Biotech) for 1–5 min. The density of the respective bands was quantified by densitometric scanning using a Hewlett-Packard scanner and Image J analyzer software (RSB). To avoid inaccuracies in protein loading, GAPDH was measured as an internal standard (Abcam, anti-GAPDH rabbit polyclonal first antibody, dilution of 1:2000; second biotinilated anti-rabbit antibody, dilution 1:2000; third streptavidin conjugated with horseradish peroxidase, dilution 1:2000) for each blot. Protein levels were calculated and expressed as the ratio between the optical densities of the bands corresponding to OCT-1, OCT-2, OCT-3, D1R and GAPDH.

### Specific Activity of Na^+^, K^+^-ATPase

Sample tissues from renal cortex weighing 50 mg were homogenized (1:10 weight/volume) in 25mM imidazole/1mM EDTA/0.25 M sucrose solution and centrifuged at 5.000 rpm and 4°C for 15 minutes. Na^+^, K^+^-ATPase activity was assayed in the supernatant using Fiske-Subbarrow method [14]. ATPase activity was measured by colorimetric determination of released orthophosphate and ouabain was used to inhibit specifically Na^+^, K^+^-ATPase activity [16]. Proteins were determined by the method of Lowry et al [15]. Results are expressed as percentage of Na^+^, K^+^-ATPase activity, considering control values as 100%.

### Dopamine assay

50-μl aliquots of urine were partially purified by batch alumina extraction, and then separated by reverse-phase high-pressure liquid chromatography (RP-HPLC) using a 4.6×250 mm Zorbax RxC18 column (DuPont Company, USA) and quantified amperometrically by the current produced upon exposure of the column effluent to oxidizing and then reducing potentials in series using a triple-electrode system (ESA, Bedford, Mass., USA). Recovery through the alumina extraction step averaged 80–90%. Dopamine concentrations in each sample were corrected for recovery of an internal standard. Levels of dopamine were further corrected for differences in recovery of the internal standard in a mixture of external standards. The limit of detection was about 20 pg/volume assayed.

### In vitro assay

Another set of rats (300–350 g) were anesthetized with 10% w/v ethyl urethane (1.3 mg/kg body weight, i.p.). Both kidneys were excised and washed with fresh Saline Krebs Buffer (SKB) to remove the residual blood. The renal cortex was dissected from the medulla by using a small scalpel. After then, slices of renal cortex were cut, minced and weighed (approximately 50 mg). ^3^H-Dopamine uptake was measured as previously described [17]. Briefly, tissues were placed in 2.0 mL SKB incubation medium in a Dubnoff incubator and pre-incubated at 37°C, pH 7.40, bubbled with a gaseous mixture of 95% O_2_ and 5% CO_2_ for 15 min; nomifensine (50 μM) was added in the medium to avoid neuronal dopamine uptake. After preincubation, tissues were transferred to fresh SKB and incubated, in similar conditions, with 0.625 μCi/mL ^3^H-dopamine (22.32 nM), 17 μM nomifensine and the different tested drugs. To determine ANP effects and signaling pathway involved in the regulation of dopamine uptake, ^3^H-dopamine uptake was measured in the absence or in the presence of D-22, anantin and KT-5823. The following groups were studied: (a) control; (b) 100 nM ANP; (c) 1 μM D-22; (d) 100 nM ANP plus 1 μM D-22; (e) 100 nM anantin; (f) 100 nM ANP plus 100 nM anantin; (g) 1 μM KT-5823; (h) 100 nM ANP plus 1 μM KT-5823.

### Statistical analysis

All values are expressed as means ± SEM and normalized respect body weight. Data was processed using Graph Pad InStat Software (San Diego, CA, USA). Statistical analysis was performed using Student’s t-test and one-way ANOVA. A value of p<0.05 was considered statistically significant.

## Results

### Renal functionality and hemodynamic parameters

The involvement of OCTs transporters in renal dopamine and ANP diuretic and natriuretic effects was investigated. To verify this objective, we determined the effects of MAO and COMT inhibition, the effects of the administration of dopamine and ANP alone, and the co-administration of ANP plus dopamine together, on renal functionality (Table 2) and on the variability of hemodynamic parameters (Table 3).

**Table 2:** Renal functional parameters of control and experimental groups.

	FENa (%)			Diuresis (μl/min/kg)			uNa.V (μEq/min/kg)			GFR (ml/min/kg)		
	Baseline	90 min	120 min	Baseline	90 min	120 min	Baseline	90 min	120 min	Baseline	90 min	120 min
**Control**	0,17±0,04	0,18±0,06	0,14±0,05	17,13±1,33	18,00±1,10	16,50±1,02	0,48±0,04	0,52±0,05	0,43±0,06	1,96±0,13	2,04±0,18	2,09±0,17
**P+T**	0,22±0,05	0,16±0,02	0,13±0,03	20,58±1,67	20,98±1,80	21,23±2,29	0,59±0,06	0,48±0,06	0,49±0,05	2,02±0,12	1,97±0,18	1,94±0,14
**ANP**	0,14±0,03	1,46±0,09*^/^^€^	1,58±0,12*^/^^€^	16,08±1,41	48,30±3,38*^/^^€^	46,79±3,22*^/^^€^	0,40±0,05	5,43±0,52*^/^^€^	5,28±0,41*^/^^€^	1,98±0,24	2,65±0,17	2,42±0,21
**DA**	0,15±0,04	1,31±0,11*^/^^€^	1,42±0,18*^/^^€^	19,14±1,65	62,10±5,21*^/^^€^	64,47±4,78*^/^^€^	0,43±0,04	4,74±0,48*^/^^€^	4,80±0,45*^/^^€^	2,07±0,28	2,52±0,15	2,36±0,18
**D-22**	0,11±0,02	0,19±0,05	0,21±0,07	16,41±4,05	17,90±2,25	20,78±3,27	0,28±0,04	0,54±0,06	0,62±0,09	1,83±0,10	2,04±0,28	2,13±0,10
**DA plus D-22**	0,12±0,06	0,20±0,05**	0,21±0,03**	16,45±1,06	28,04±1,31**	29,40±1,39**	0,36±0,04	0,80±0,04**	0,78±0,40**	1,97±0,19	2,62±0,11	2,43±0,25
**ANP plus D-22**	0,23±0,10	1,16±0,20*^/^^€^	1,28±0,20*^/^^€^	20,24±3,08	35,52±2,54*^/^^€^	36,89±4,57*^/^^€^	0,68±0,23	3,67±0,34*^/^^€^	3,48±0,48*^/^^€^	2,10±0,10	2,20±0,11	1,89±0,12
**ANP plus DA**	0,16±0,04	2,45±0,15**^/^***^/^^€^	2,22±0,16**^/^***^/^^€^	17,34±1,50	92,26±7,17^$^^/^^€^	95,24±8,56^$^^/^^€^	0,44±0,07	10,43±0,85^$^^/^^€^	9,95±1,01^$^^/^^€^	1,92±0,22	3,17±0,27*^/^^€^	3,02±0,16*^/^^€^
**ANP plus DA plus D-22**	0,19±0,03	1,56±0,10^#^^/^^€^	1,64±0,11^#^^/^^€^	18,08±1,03	54,41±4,98^#^^/^^€^	52,20±4,22^#^^/^^€^	0,50±0,03	5,65±0,70^#^^/^^€^	5,80±0,62^#^^/^^€^	2,09±0,23	2,56±0,24	2,52±0,29

^€^p<0.05 vs respective baseline period;

*p<0.05 vs Control or P+T groups;

**p<0.05 vs DA;

***p<0.05 vs ANP;

^#^p<0.05 vs ANP plus DA;

^$^p<0.05 vs DA or ANP.

**Table 3:** Hemodynamic parameters of control and experimental groups.

	MAP (mmHg)			HR (beats/min)		
	Baseline	90 min	120 min	Baseline	90 min	120 min
**Control**	82±4	84±5	82±4	390±10	384±11	374±9
**P+T**	83±5	85±3	83±4	400±8	386±12	374±13
**ANP**	78±5	73±3	70±4	388±12	368±8	366±11
**DA**	79±4	75±4	71±3	382±13	374±13	380±10
**D-22**	79±5	80±5	79±2	390±9	370±4	365±10
**DA plus D-22**	78±5	76±3	75±3	378±13	370±10	368±9
**ANP plus D-22**	74±4	78±5	69±5	385±9	370±19	376±22
**ANP plus DA**	80±4	63±5*	61±4*	382±9	412±11**	420±12**/^€^
**ANP plus DA plus D-22**	79±4	70±4	69±5	370±8	366±12	380±10

MAP: Mean arterial pressure; HR: Heart rate.

*p<0.05 vs Control and P+T groups;

**p<0.05 vs ANP;

^€^p<0.05 vs Control, P+T, ANP and DA plus D-22 groups

To investigate the participation of OCTs, D-22 was used as specific inhibitor of these transporters. Inhibition of MAO and COMT (P+T group) did not alter any renal functional or hemodynamic parameter compared to control rats (Tables 2 and 3). Under MAO and COMT inhibition, the infusion of either ANP or dopamine alone increased the diuresis and the fractional and urinary excretion of sodium significantly compared with their respective baseline levels, as well as *versus* control and P+T groups (Table 2). The addition of D-22 alone did not modify any renal functional or hemodynamic parameter when compared to control or P+T rats (Tables 2 and 3). However, the natriuretic and diuretic effects elicited by the infusion of dopamine (DA group) were prevented by D-22 administration (DA plus D-22 group). On the other hand, the administration of ANP plus dopamine achieved a greater response on the diuresis and the fractional and urinary excretion of sodium that equaled the sum of ANP and dopamine effects (additive effect rather than potentiation), when compared with ANP or dopamine treatment alone. Renal and hemodynamic effects elicited by ANP were not prevented by the administration of D-22 (Tables 2 and 3). However, under simultaneous co-infusion of ANP and dopamine (ANP plus DA plus D-22 group), the administration of D-22 diminished the additive effect elicited by both agents on natriuresis and diuresis, although the values did not reach baseline levels and they were not significantly different from those of ANP group. The GFR increased only in the ANP plus dopamine group with respect to their baseline values and compared with control and P+T groups respectively, while it remained unchanged in the rest of the experimental groups (Table 2). MAP and heart rate values were not altered in any experimental group over 120 minutes, except in ANP plus dopamine group where MAP values decreased relative to their baseline levels and compared with control and P+T groups (Table 3).

### Specific activity of Na^+^, K^+^-ATPase

The involvement of OCTs transporters in the inhibitory effects of renal dopamine and ANP on Na^+^, K^+^-ATPase specific activity was investigated. To quantify this, Na^+^, K^+^-ATPase specific activity was determined in samples of renal cortex obtained at the end of the experimental period.

**Fig 1:**
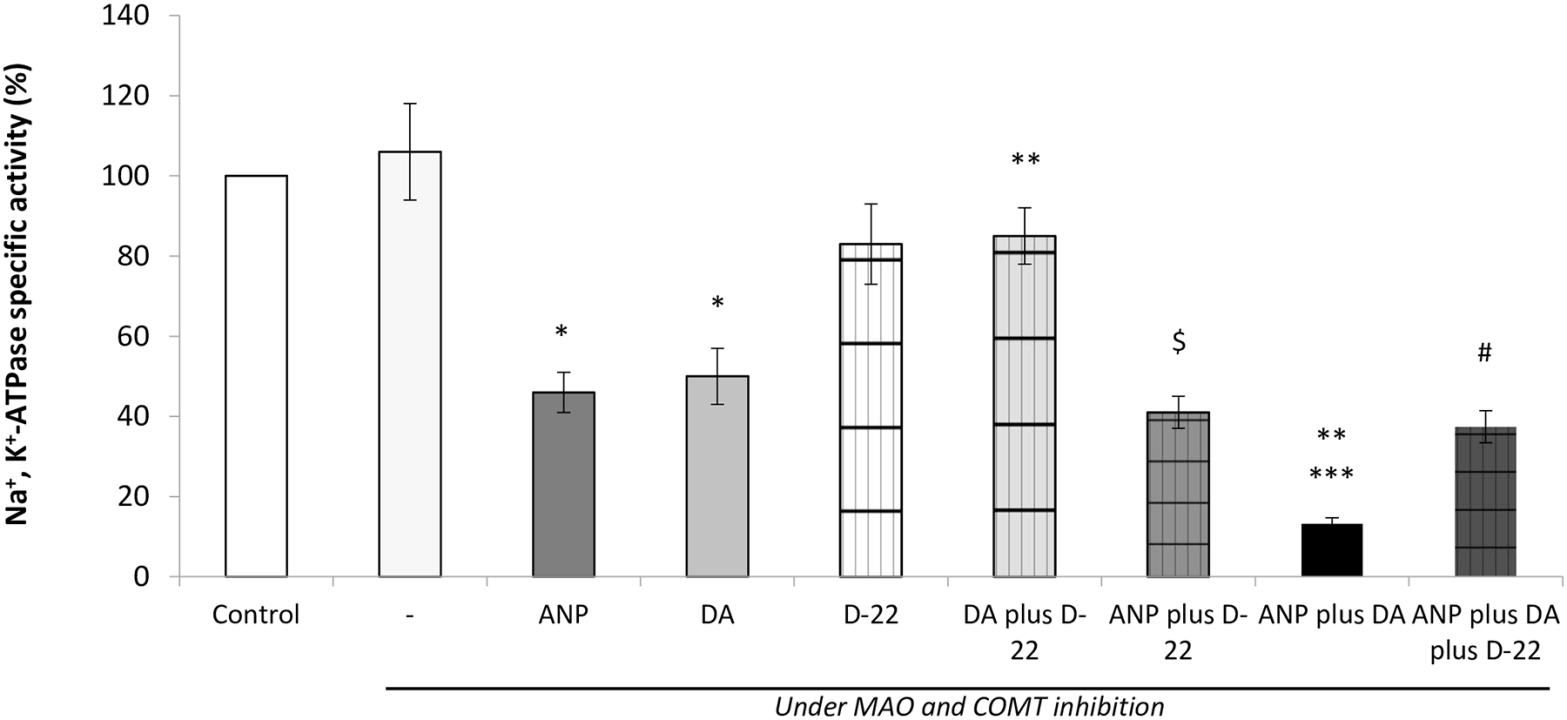
In vivo inhibition of OCTs with D-22 and its effects on Na^+^, K^+^-ATPase activity in the presence of dopamine (DA) and ANP plus DA. *p<0.001 vs Control/P+T; **p<0.05 vs DA; ***p<0.05 vs ANP; ^#^p<0.05 vs ANP plus DA; ^$^p<0.05 vs D-22.

In accordance with previous studies [14], the infusion of either ANP or dopamine decreased Na^+^, K^+^-ATPase activity by approximately 56% and 53% respectively (Fig 1). To evaluate the participation of OCTs, we used D-22 as specific blocker of these transporters. D-22 did not affect Na^+^, K^+^-ATPase activity by itself, but reversed the inhibitory effect of dopamine on the enzymatic pump. The co-infusion of ANP plus dopamine increased the inhibition (by 87%) of Na^+^, K^+^-ATPase activity elicited by ANP or dopamine alone. On the other hand, the addition of D-22 partially reversed the inhibition produced by the co-administration of ANP plus dopamine, reaching values very similar to those registered with the infusion of ANP or dopamine alone.

### Urinary dopamine excretion

**Fig 2:**
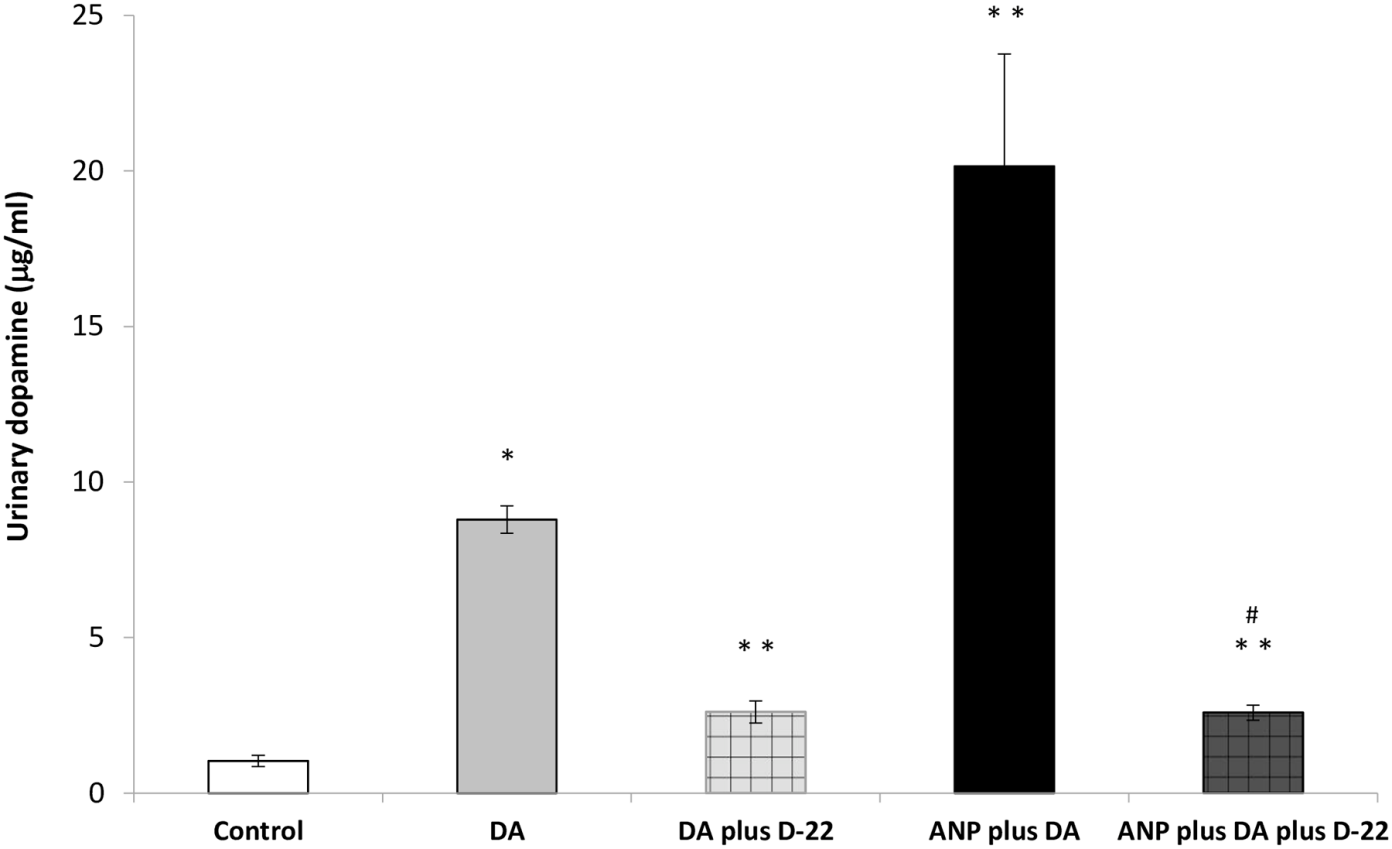
Effects of exogenous dopamine, ANP and D-22 on dopamine urinary levels. *p<0.05 vs Control; **p<0.05 vs DA; ^#^p<0.05 vs ANP plus DA.

The possibility that the inhibition of OCTs may affect the stimulatory effects of ANP on dopamine tubular transport or may alter dopamine urinary excretion was evaluated. To quantify this, dopamine excretion was determined in urine during the last experimental period. Because of the administration of benserazide, almost all urinary dopamine was considered to be from the infusion of exogenous dopamine. As expected, the exogenous infusion of dopamine significantly increased the excretion of the amine when compared with the control group. This increase was inhibited by the administration of D-22. The co-infusion of ANP and dopamine together further increased dopamine urinary excretion even compared with dopamine alone group. This increase was also inhibited by D-22, reaching very similar urinary dopamine levels to those observed in control group (Fig 2).

### Western blot analysis of OCTs and D1 receptor protein expression in renal cortex

**Fig 3:**
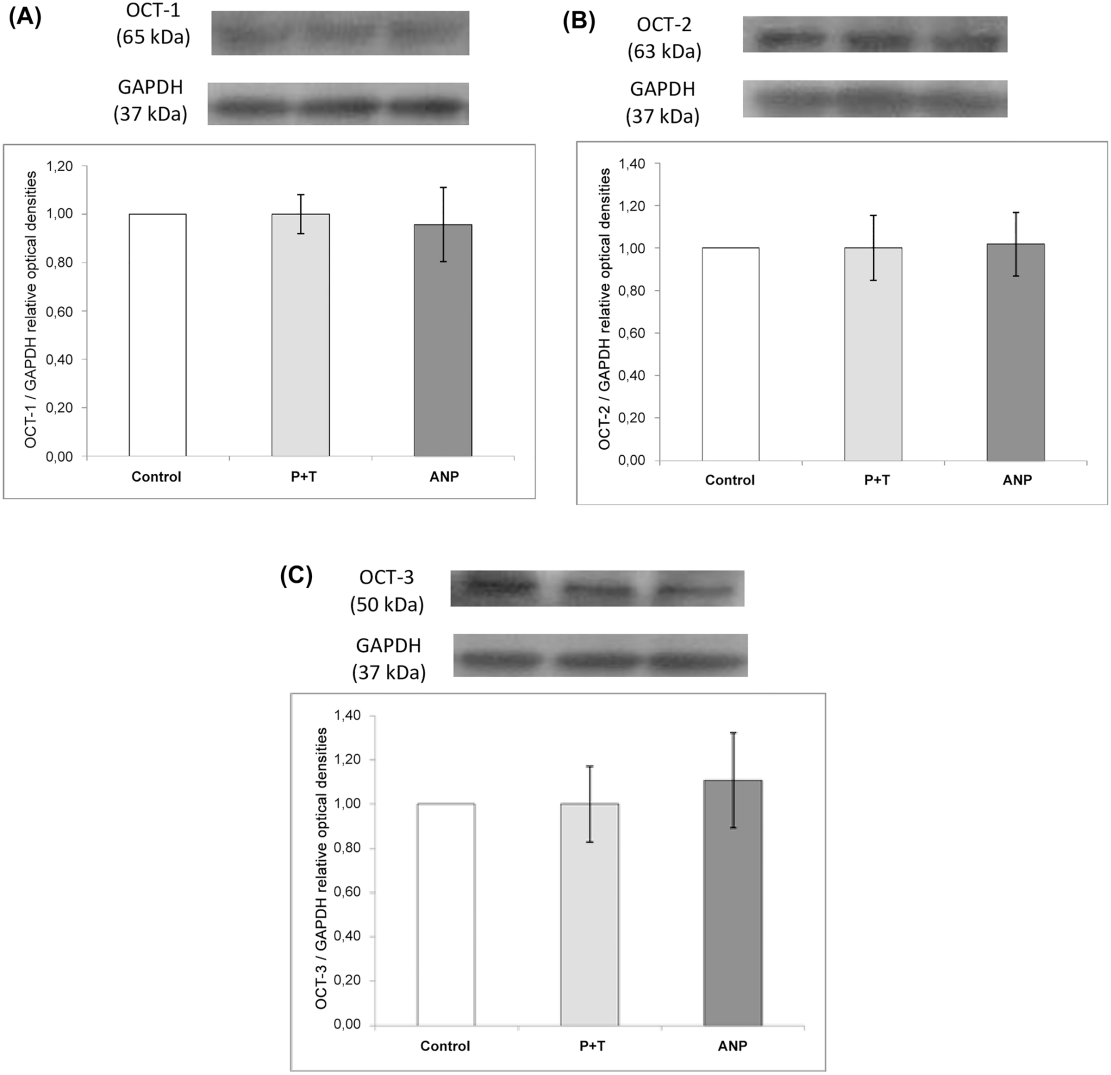
A, B and C. Western blot analysis of OCT-1 (A), OCT-2 (B) and OCT-3 (C) in renal cortex: Effects of MAO and COMT inhibition by pargyline and tolcapone (P+T) and ANP infusion on OCT-1 (A), OCT-2 (B) and OCT-3 (C) protein expression in membrane preparations from renal cortex. Histograms illustrate the values of protein expression of OCTs in each group, normalized to GAPDH expression.

Based on the above results, we tested the possibility that ANP might stimulate the protein expression of OCTs and/or dopamine D1R at membrane level, and consequently, the fact that the natriuretic peptide could increase the urinary excretion of dopamine and enhance its natriuretic effects. To assess this possibility, western blot analysis of OCTs and D1R were performed on membrane preparations from renal cortex tissues. Samples were probed with anti-OCT-1, anti-OCT-2, anti-OCT-3 and anti- D1R antibodies and quantified using GAPDH as an internal standard. Fig 3A, 3B and 3C show that neither MAO and COMT inhibition nor ANP infusion altered renal OCT-1, OCT-2 or OCT-3 protein expression compared with control group.

**Fig 4:**
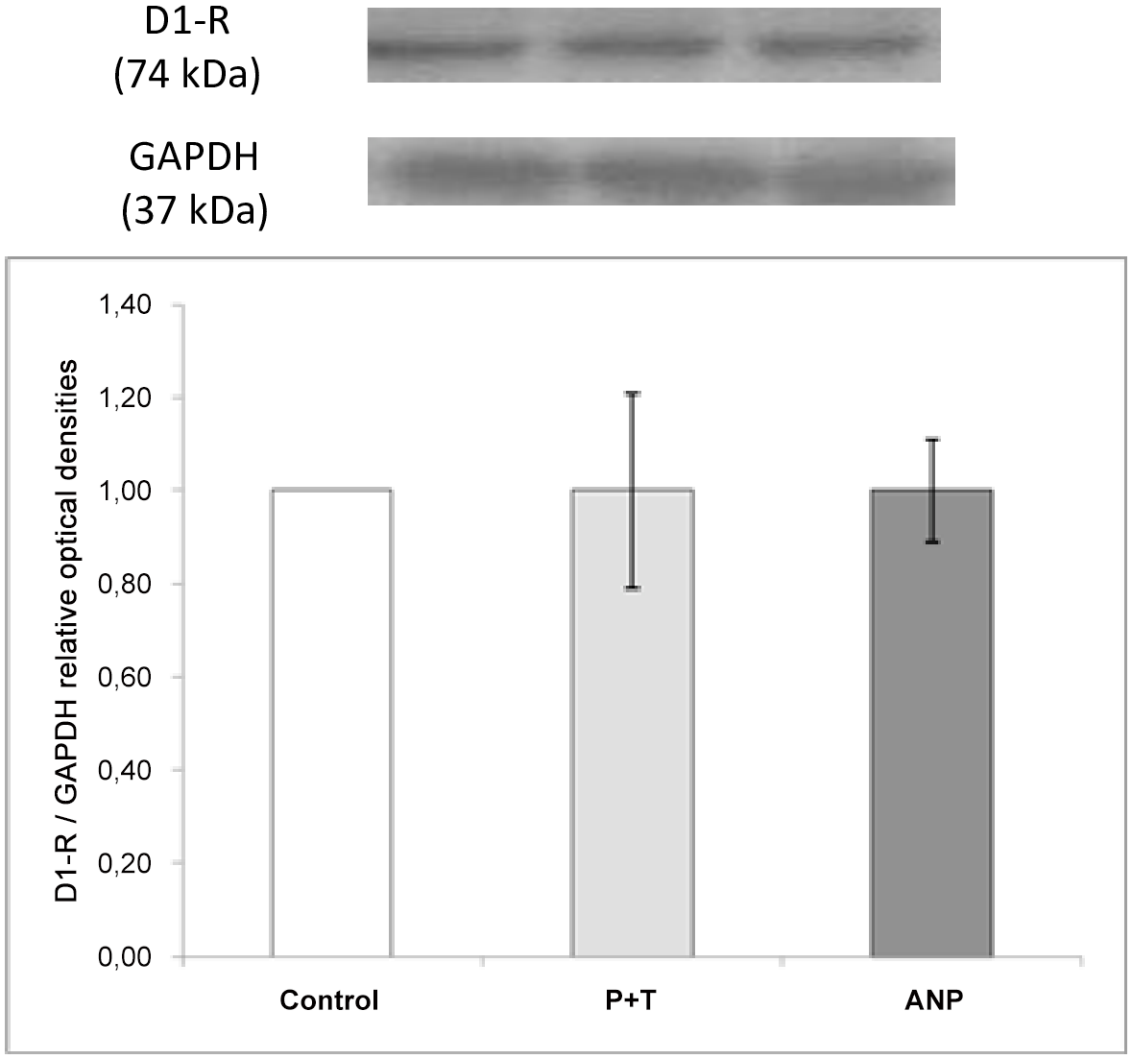
Western blot analysis of D1R in renal cortex: Effects of MAO and COMT inhibition by pargyline and tolcapone (P+T) and ANP infusion on D1R expression in membrane preparations from renal cortex. Histograms illustrate the values of protein expression of D1R in each group, normalized to GAPDH expression.

Although difference in expression did not reach statistical significance, a trend towards to increase OCT-3 expression can be observed in the ANP group. In Fig 4, it can be observed that neither MAO and COMT inhibition nor ANP infusion altered renal dopamine D1R protein expression when compared with control group.

### In vitro effects of ANP on ^3^H-dopamine uptake assay

**Fig 5:**
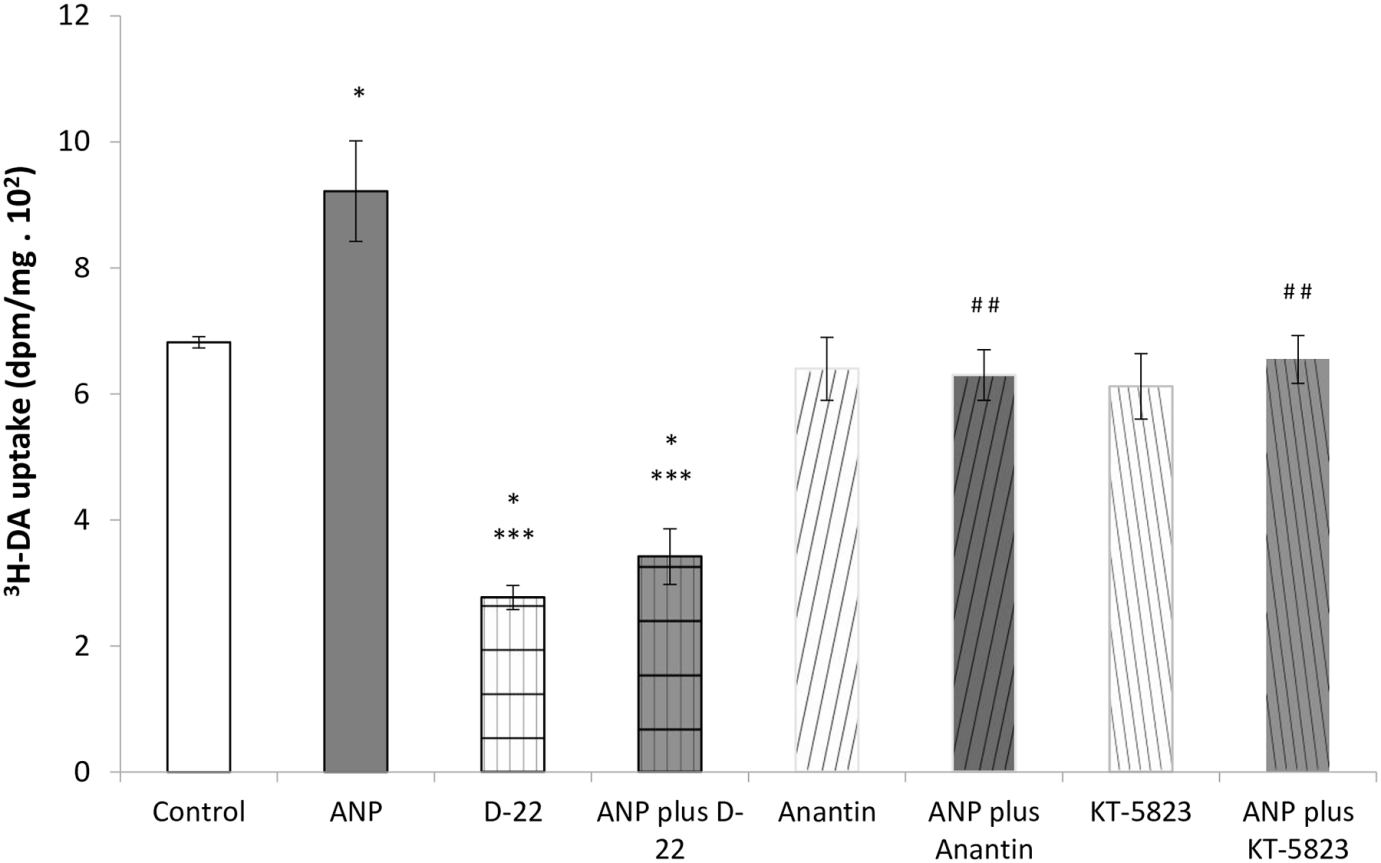
Effects of ANP, D-22, anantin and KT 5823 on ^3^H-dopamine uptake. *p<0.05 vs Control; ***p<0.01 vs ANP; ^##^p<0.05 vs ANP.

In order to evaluate whether the increased urinary excretion of dopamine elicited by ANP administration is caused by stimulation of dopamine tubular transport by OCTs, we carried out an in vitro assay in renal cortex slices using ^3^H-dopamine. Fig 5 demonstrates that 100 nM ANP significantly increased ^3^H-dopamine uptake (approximately 35%) in renal cortex slices compared with control group. In presence of D-22 (1μM), ^3^H-dopamine uptake was significantly reduced by approximately 60%. As demonstrated in Fig 5, the administration of D-22 completely abolished the stimulating effect of ANP on renal dopamine uptake. In addition, the intracellular signaling pathway involved in the stimulating effect of ANP on dopamine uptake was explored. It is well known that most of renal actions of ANP are mediated by NPR-A receptors, leading to an increase in intracellular cGMP generation and activation of protein kinase G (PKG) [18,19]. Therefore, we carried out another set of experiments in kidney slices in order to evaluate ANP signaling pathway by using anantin (NPR-A specific blocker) and KT-5823 (specific inhibitor of PKG). As demonstrated in Fig 5, 100 nM anantin alone did not alter ^3^H-dopamine uptake by itself, but when added together with ANP, it was able to abolish the increased dopamine uptake elicited by the natriuretic peptide. Moreover, the specific inhibitor of PKG, KT-5823 (1 μM) reversed ANP stimulatory effects on dopamine uptake. On the other hand, KT-5823 by itself did not alter ^3^H-dopamine uptake.

### Discussion

We have previously reported that ANP stimulates dopamine uptake in renal cortex, this process being characterized as a typical extraneuronal uptake [17]. In our in vivo protocols, we used benserazide to inhibit endogenous dopamine synthesis and in order to avoid interfering with the effects of exogenous dopamine. Considering that MAO and COMT are capable of catabolizing renal tubular dopamine, we inhibited the catabolism of infused exogenous dopamine by administrating P+T, respectively, to all experimental groups before the beginning of the experimental period. When compared with control group, administration of P+T did not modify the diuresis, natriuresis and GFR, as well as cardiovascular parameters. Therefore, we decided to compare all results from the rest of the experimental groups with P+T group. Our results confirmed that the administration of ANP (5 μg/kg i.v. bolus plus 10 μg/kg i.v. infusion) or dopamine (100 μg/kg i.v. infusion) alone exerted natriuretic and diuretic properties, without affecting the GFR. In addition, the co-infusion of ANP and dopamine further increased the diuresis and natriuresis, exhibiting an additive effect on their renal actions when compared with the infusion of ANP or dopamine alone. On the other hand, in vivo administration of the specific inhibitor of OCTs, D-22, was able to inhibit the stimulation of urine output as well as urinary and fractional sodium excretion elicited by exogenous dopamine, demonstrating that the OCTs are essential carriers for dopamine to exert its renal effects. In addition, D-22 significantly reduced the effect of ANP plus dopamine administration, reaching water and sodium excretion values very similar to those obtained with infusions of ANP or dopamine alone (Table 2). Since some creatinine is actively secreted via OCTs, the administration of D-22 could reduce the apparent creatinine clearance without affecting the true GFR. Nonetheless, in our experiments, creatinine clearance was not affected in the presence of D-22 alone or when this blocker was administered together with ANP or dopamine. These evidences point out that, when OCTs activity is blocked, other tubular transporters, like organic anion transporters (OAT), may be responsible for creatinine secretion. However, further experiments involving OATs blocking must be performed to confirm this hypothesis.

The identification of a dopamine specific tubular transporter is of great interest for basic and clinical research since it would allow insight into the mechanisms that affect the exogenous dopamine availability in the tubular lumen and therefore its natriuretic and diuretic efficacy. The clinical efficacy of dopamine infusion to prevent renal failure (e.g. patients with pre existing renal disease or at risk for developing it, as is the case in heart failure) has been questioned by a number of clinical studies [20–23]. Several studies indicate that exogenous dopamine has no effect on promoting renal sodium and water excretion in pathological conditions [24,25]. This lack of action could be related, among other causes, to an alteration in dopamine tubular transport from the bloodstream into the tubular lumen, affecting its bioavailability to interact with D1 and D2 receptors. Since almost 95% of the dopamine located in the bloodstream is sulfo-conjugated, and therefore cannot cross the glomerular barrier, exogenous dopamine needs to be uptaken from the basolateral side of renal tubules to reach the apical luminal side where dopamine receptors are available [26]. Given their location, OCTs seem to be the tubular transporters responsible for mediating dopamine translocation from bloodstream into the cellular compartment [27]. Through this mechanism, D-22 significantly reduces the tubular secretion of dopamine while it increases its spillover into systemic circulation [28–30]. The results of our study prove that the integrity of OCTs activity is necessary not only to elicit the diuretic and natriuretic actions of dopamine, but also for ANP to exert its full diuretic and natriuretic effects. To our knowledge this is the first in vivo report to show that inhibition of OCTs can alter the natriuretic and diuretic response to the exogenous administration of dopamine as well as the co-administration of ANP plus dopamine.

Our next step was to test in vivo the possibility that the inhibition of OCTs by D-22 could reduce the inhibitory action of exogenous ANP and dopamine on Na^+^, K^+^-ATPase specific activity. As expected, either ANP or exogenous dopamine inhibited renal specific activity of Na^+^, K^+^-ATPase, an enzyme closely linked to water and sodium retention. Moreover the co-infusion of ANP plus dopamine increased further pump inhibition, showing an enhanced response. On the other hand, the inhibition of OCTs by D-22, prevented the inhibition of Na^+^, K^+^-ATPase activity caused by dopamine alone and also reduced the inhibition caused by dopamine plus ANP.

In order to confirm the stimulatory effect of ANP on dopamine tubular transport, mediated by OCTs, we proceeded to test whether the urinary excretion level of dopamine could be altered by the presence of ANP and/or D-22. This experiment allowed us to discard a simple addition of independent effects of both natriuretic agents. Our results indicate that the inhibition of OCTs by D-22 can decrease urinary dopamine excretion observed after the infusion of exogenous dopamine. This supports the fact that the administration of D-22 reduced Na^+^, K^+^-ATPase inhibition, as well as water and sodium excretion, both stimulated by dopamine. Moreover, the co-infusion of ANP and dopamine further increased urinary dopamine levels when compared with dopamine group, and this increase was also blocked by inhibition of OCTs by D-22. These results clearly suggest that ANP promotes the uptake of exogenous dopamine by the renal tubules into the tubular lumen through stimulation of OCTs synthesis and/or their specific activity. These results also correlate with our findings that the co-infusion of ANP and dopamine further increase water and sodium excretion by the kidney as well as inhibiting Na^+^, K^+^-ATPase activity. We have previously demonstrated that ANP and urodilatin stimulate dopamine uptake in renal cortex, while angiotensin II exerts the opposite effect [17,31,32]. On the other hand, Carranza et al. have demonstrated that another hormone, insulin, can stimulate L-dopa uptake into proximal tubular cells [33]. In line with this finding, in insulin-resistance models with fructose overload during 4 weeks, proximal tubular cells exhibit lower renal L-dopa reabsorption as compared to control animals and thereby increasing L-dopa/dopamine plus 3,4-dihydroxyphenylacetic acid (DOPAC) ratio [34,35].

The effects of ANP on OCTs may be the result of an increase in their protein expression and/or activity. The protein expression of both, OCT-1 and OCT-2, is reduced in experimental model of diabetes induced by streptozotocin in rats, thereby affecting the transport of a large number of exogenous and endogenous compounds [36]. On the other hand, it has been reported that the phosphorylation of OCTs across the basolateral membrane can modify their activity as transporters [37,38]. In HEK293 cells that express rat OCT-1, the activity of this transporter is stimulated by proteinkinase C and proteinkinase A, while it is inhibited by cGMP [39,40]. ANP might stimulate the expression of OCTs and, in consequence, could increase the urinary excretion of dopamine. On the other hand, to date there are no studies that address whether or not ANP can stimulate OCT-1, OCT-2 or OCT-3 protein expression in renal tissue. In the present study, protein expression of three OCTs tested was not altered by ANP treatment, although a non-significant trend towards increased expression of OCT-3 was seen. These results should not be considered definitive, taking into account that the experimental period may have been too short to reveal a significant change in OCTs protein synthesis. However, in this brief experiment, we preferred to measured the protein content of OCTs in isolated membrane by western blot, because this data gives a more accurate indication of the available and functional OCTs to transport dopamine.

As it was previously mentioned, even if ANP did not alter OCTs expression, the fact that ANP enhances OCTs activity may provide another way to temporarily regulate the dopamine tubular transport by OCTs. To test whether the increase in urinary dopamine excretion observed after ANP infusion could be related to the stimulation of OCTs activity by ANP, we performed an in vitro experiment in renal cortex slices incubated with ^3^H-dopamine. Fig 5 shows that ANP stimulates ^3^H-dopamine uptake (by approximately 35%) over a short time period, in renal cortex as compared with the control group. OCTs inhibition by D-22 reduced ^3^H-dopamine uptake compared with control group by approximately 60%. Once again, this result demonstrates that OCTs are specific transporters for dopamine, and their activity is critical in the process of dopamine uptake by the kidney. On the other hand, even in the presence of ANP, D-22 decreased ^3^H-dopamine uptake by 50% compared with control group, reinforcing the idea that ANP stimulates dopamine tubular uptake by increasing OCTs activity. The enhancing effects of ANP on dopamine uptake are mediated by stimulation of NPR-A receptors and PKG activation, since anantin (specific blocker of NPR-A receptor) and KT-5823 (specific inhibitor of PKG) reverse ANP stimulatory effects on dopamine uptake in kidney slices. Moreover, ANP promotes the urinary excretion of dopamine by stimulating OCTs activity. The analysis of all these results as a whole suggests that the secretion of dopamine into the tubular lumen would allow D1R activation and Na^+^, K^+^-ATPase activity inhibition, leading to decreased sodium reabsorption and increased natriuresis. By these mechanisms, ANP and dopamine may regulate and enhance natriuresis and diuresis through a common pathway.

Under physiological conditions, the natriuretic and diuretic effects elicited by dopamine are exerted mainly by D1-like receptors [14]. Aperia has reported homologous sensitization of D1R in rat renal cortical slices, thereby increasing its availability to luminal dopamine [3]. In addition, Holtbäck et al. demonstrated that ANP can also recruit D1R to the plasma membrane (heterologous sensitization of D1R), facilitating D1R stimulation by dopamine [41]. ANP might also stimulate the expression of D1R and, in consequence, the natriuretic peptide could increase the natriuretic and diuretic effects elicited by dopamine. To assess this possibility, the expression levels of D1R were measured by western blot in membrane isolated homogenates of renal cortex tissues. As shown in Fig 4, there were no significant changes in D1R expression in the presence of ANP. Therefore, the additional effects observed after ANP and dopamine co-infusion on renal functional parameters and Na^+^, K^+^-ATPase activity are due to the stimulatory effects of ANP on exogenous dopamine OCTs-dependent transport into the tubular lumen.

**Fig 6:**
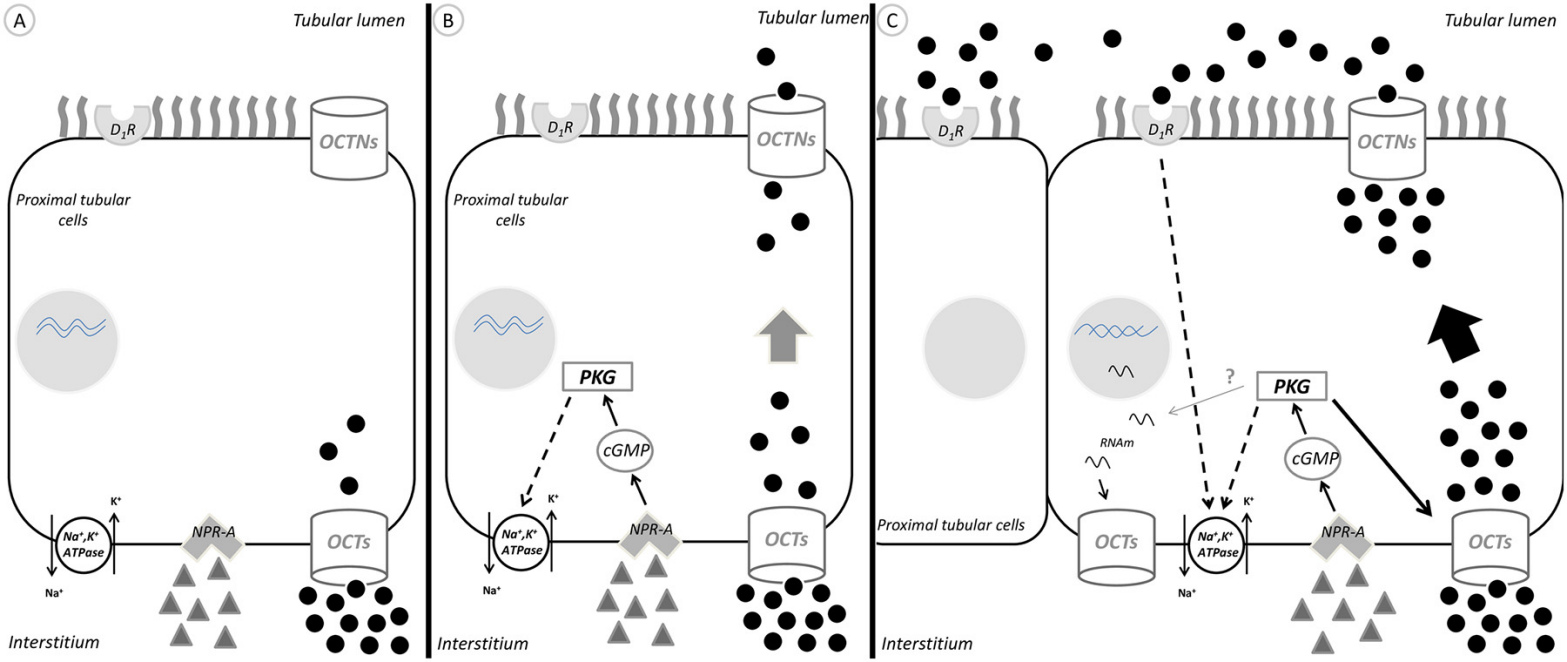
Schematic representation of the mechanism by which ANP could enhance dopamine tubular transport in proximal tubule cells, by stimulation of organic cationic transporters. A: under basal conditions, exogenous dopamine is carried by OCTs located at the basolateral membrane and uptaken from circulation and interstitium by the proximal tubular cells. B: Once inside the tubular cell, dopamine can reach the tubular lumen through the OCTNs located at the apical membrane. Atrial natriuretic peptide inhibits Na^+^, K^+^-ATPase activity through stimulation of NPR-A receptors, cGMP and PKG. C: ANP enhances dopamine tubular transport by OCTs, stimulating its specific activity, and increasing dopamine concentration at the luminal side. The simultaneous inhibition of Na^+^, K^+^-ATPase activity by ANP and dopamine promotes a greater natriuretic response. OCTs: organic cationic transporters. OCTNs: carnitine/organic cationic transporters. Black circles: dopamine; gray triangles: ANP. Full arrows: stimulation; dot arrows: inhibition; gray arrow with?: hypothetical mechanism.

This work provides evidence of a novel mechanism by which ANP acts together with dopamine to enhance sodium and water excretion by increasing dopamine tubular bioavailability (Fig 6).

A failure in the mechanism of dopamine transport in renal tubule cells, associated to changes in OCTs activation or expression, could be postulated as a cause of sodium and water retention and contribute to the development of hypertension. Moreover, this mechanism could explain the lack of effects observed in clinical studies in which exogenous dopamine infusion is not capable of preventing the impairment of renal function in patients with acute renal injury [22,24]. In most of these studies, patients recruitment includes those with chronic renal disease (CRD) or a condition which increases the risk for CRD, a clinical context where OCTs functional status is unknown [22,24,25]. Similarly, in most of the clinical studies the urinary excretion of dopamine was not measured. This is unfortunate as this data could be used as a direct marker of the amount of dopamine infused that reaches the luminal side of renal tubules and therefore is available to stimulate D1R.

Together, these results highlight the role of OCTs as transporters of dopamine in the kidney, the relationship between ANP and dopamine to enhance each other´s natriuretic and diuretic properties, and the importance of OCTs as an essential factor for dopamine to exhibit its full natriuretic and diuretic effects. Therefore it may be hypothesized that the lack of clinical benefits reported after dopamine infusions could be related to an alteration in its transport to the lumen side by changes in OCTs activity. Further experimental and clinical studies must be done to confirm this hypothesis.

## Conclusion

In summary, this study demonstrates a novel mechanism for how ANP increases dopamine levels at the tubular lumen by stimulation of dopamine uptake by OCTs located at the basolateral side of tubular proximal cells. This effect increases the availability of dopamine to interact with D1R located at the luminal side of tubular cells. Through this mechanism, both natriuretic agents enhance their effects to promote sodium and water excretion by the kidneys.
